# How Do Pharmacists Develop into Advanced Level Practitioners? Learning from the Experiences of Critical Care Pharmacists

**DOI:** 10.3390/pharmacy5030038

**Published:** 2017-07-07

**Authors:** Ruth E. Seneviratne, Helen Bradbury, Richard S. Bourne

**Affiliations:** 1 Departments of Pharmacy and Critical Care, Sheffield Teaching Hospitals, Northern General Hospital, Herries Road, Sheffield S5 7AU, UK; Richard.bourne@sth.nhs.uk; 2 Faculty of Medicine and Health, University of Leeds, Leeds LS2 9JT, UK; h.m.bradbury@leeds.ac.uk

**Keywords:** advanced practice, pharmacy, education

## Abstract

The national UK standards for critical care highlight the need for clinical pharmacists to practise at an advanced level (equivalent to Royal Pharmaceutical Society, Great Britain, Faculty Advanced Stage II (MFRPSII)) and above. Currently the UK is unable to meet the workforce capacity requirements set out in the national standards in terms of numbers of pharmacist working at advanced level and above. The aim of this study was to identify the strategies, barriers and challenges to achieving Advanced Level Practice (ALP) by learning from the experiences of advanced level critical care pharmacists within the UK. Eight participants were recruited to complete semi-structured interviews on their views and experiences of ALP. The interviews were analysed thematically and three overarching themes were identified; support, work-based learning and reflective practice. The results of this study highlight that to increase the number of MFRPSII level practitioners within critical care support for their ALP development is required. This support involves developing face-to-face access to expert critical care pharmacists within a national training programme. Additionally, chief pharmacists need to implement drivers including in house mentorship and peer review programmes and the need to align job descriptions and appraisals to the Royal Pharmaceutical Society, Great Britain, Advanced Practice Framework (APF).

## 1. Introduction

Hospital pharmacy practice in the UK has developed significantly over the last 10 years with the recognition of different levels of professional practice with an assessment process [1,2]. In 2005, three levels of pharmacy practice were defined by the Competency Development and Evaluation Group:- General or Foundation level, Advanced Level Practice (ALP) and Mastery or Consultant level [1]. The United Kingdom Clinical Pharmacy Association (UKCPA) has been involved in leading the development of professional recognition with the UKCPA Critical Care Group (CCG) being instrumental in developing a recognition of practice assessment. Firstly, they established a competency framework for Foundation level and ALP alongside a syllabus [3] specifically for critical care pharmacists [4]. Subsequently they created a credentialing process to assess critical care pharmacists for ALP [5]. 

The credentialing or professional recognition assessment developed by the UKCPA CCG has now been superseded by the Royal Pharmaceutical Society (RPS) Faculty [2]. The RPS has recognised three new levels of practice; Advanced Faculty Stage I (MFRPSI), Advanced Faculty Stage II (MFRPSII) considered equivalent to ALP, and Faculty Fellow (FFRPS) [2]. Pharmacists working within any speciality can be assessed for any of the three levels of professional recognition [2]. The RPS Faculty assessment comprises of a practice-based portfolio, peer assessment and expert practice assessment [2]. These assessments are underpinned by the Advanced Pharmacy Framework (APF) which has been developed from the UKCPA CCG’s competency framework [6]. 

For pharmacists working within critical care the UK standards for intensive care units highlight the requirement for pharmacists to be working at, or have access to a pharmacist at ALP level and above [7,8]. Although these ALP standards have been in place for several years, a recent comprehensive UK workforce survey of 410 critical care pharmacists emphasised that only half are working at advanced level or above [9]. Moreover, of those non-ALP pharmacists approximately a third did not have access to an ALP pharmacist at their institution [9]. Warin et al. [10] surveyed critical care pharmacists and found they felt that there were no suitable training programmes to help them to develop ALP. The key recommendation from the survey was a requirement to develop a national or regional ALP training programme [10]. In response to this recommendation the UKCPA led a project to identify an appropriate model for an ALP education and training programme using critical care as an example of specialist pharmacy practice. This study formed a part of the project and focused and on identifying the strategies, barriers and challenges to achieving ALP by learning from the experiences of advanced level critical care pharmacists. Specifically, the study objectives were to identify:

1. The factors that advanced level critical care pharmacists perceive to facilitate or hinder them in developing ALP
2. How advanced level critical care pharmacists have overcome the barriers and challenges to developing ALP
3. The strategies advanced level critical care pharmacists have drawn upon to develop ALP
4. The implications for future education provision for ALP

## 2. Methods

In order to explore the experiences of critical care pharmacists a phenomenological approach was employed [11,12]. There were twenty-one critical care pharmacists that had undertaken the UKCPA credentialing process and have been officially recognised as practising at advanced level. These twenty-one critical care pharmacists were sent an email to invite them to volunteer to take part in this study. Eight participants responded and were recruited to complete semi-structured interviews on their views and experiences of ALP. Guest et al. [13] states that six to twelve interviews is an adequate number when using a phenomenological approach to allow themes and conclusions to be drawn from the data. Therefore, it was felt that eight participants would be a representative sample.

The interview questions were informed by the study objectives and a literature review. They were designed to capture the strategies, barriers and challenges faced by the participants in developing their practice. In particular, the study focused on the influence of the work environment and how gaps in knowledge and professional development were identified. In addition, they were asked to provide advice for pharmacists developing their practice to advanced level allowing them to consider the current challenges faced (Appendix A: Interview schedule). A pilot interview was undertaken which enabled the appropriateness of the questions to be reviewed and this completed interview was included in the analysis. The interviews were conducted from October to December 2015. Six of the participants were met face-to-face and a further two participants were interviewed via video conferencing.

The interviews were audio recorded, fully transcribed and then analysed thematically. The analysis used both an inductive approach, allowing the themes to emerge from the interview data itself and a deductive approach, using the interview questions and study objectives to verify the themes [12]. The individual interview transcripts were reviewed and the important quotes were highlighted and then annotated with descriptive codes [11]. Descriptive codes were used to identify and describe the relevant aspects of the participants’ views, experiences and perceptions [13]. This process was repeated confirming the original codes and by adding new descriptive codes until a saturation point was reached where no new codes could be identified [14,15]. Interpretative codes were drawn from the descriptive codes by focusing on defining the meaning behind the participants’ views, experiences and perceptions [14]. The data was then further revised and refined by looking at contrasting interviews:- women compared to men, experienced (≥10 years in critical care) with less experienced (<10 years in critical care) and working within a teaching hospital compared with a district general and by looking at each individual interview question [14]. This allowed the interpretive codes to be further revised, refined and grouped together to reveal basic themes [14].

## 3. Ethics

The study received ethical approval from the University of Leeds, Research Ethics Committee on 28 September 2015 (Reference number: LTEDUC-074). The participants gave informed consent and were able to withdraw from the process up to the time of analysis. To ensure the anonymity of the individuals interviewed the transcripts were reviewed and text removed that could identify individuals such as the reference to their place of work or names of work colleagues.

## 4. Results

The thematic analysis identified three overarching themes. These were support, work-based learning and reflective practice. The main themes were then explored by considering what they signified for critical care pharmacy and critically reviewed with the appropriate literature. The eight participants recruited were of different genders (four male, four female), had different levels of experience (five experienced, three less experienced), were based in different UK locations and included experiences of working in teaching (six participants) and district general hospitals (two participants). There appeared to be no significant differences between the male and female participants’ views but there were some differences between those who worked in teaching hospitals and district generals and those who were experienced compared to those less experienced. 

### 4.1. Support

The critical care pharmacists described receiving support in the form of mentorship and supervision.

#### 4.1.1. Mentorship

Critical care pharmacists valued the role of mentors and found it easy to seek out mentorship both inside and outside of their workplace. In the workplace mentors were medical professionals and pharmacists. These pharmacists worked both within critical care, if they were part of a critical care pharmacy team, and in different specialities. Mentorship was also found outside of the workplace usually from critical care pharmacists whom they linked with through the UKCPA online discussion boards, at meetings or by arranging visit to other units. However, mentorship arrangements were informal and relied on the generosity of other practitioners.

“With regard to developing my advanced practice, I joined the UKCPA critical care expert group and I started to get email support either on an individual question basis or just being able to contact or speak to people at meetings.”

*(Participant 1, teaching hospital, experienced)*

In some areas of the UK the pharmacists described local critical care pharmacy networks that organised education and training sessions, offered support, advice and sharing of best practice. A couple of pharmacists discussed the importance of their local network in supporting critical care pharmacists to develop ALP. However, there was felt to be some variability in the provision of these critical care pharmacy networks within the UK. Some areas did not have an active network and pharmacists working in these localities were felt to be at a disadvantage. The interviewees that worked as lone practitioners felt strongly that there was a need for access to other critical care pharmacists to help them to develop their practice.

#### 4.1.2. Supervision

The pharmacists described a lack of formal work-based supervision but identified its importance for developing certain areas of the ALP framework. Formal supervision was only provided whilst undertaking an academic qualification or when working in a critical care pharmacy team. When undertaking academic qualifications such as a research masters, supervision was usually provided by a senior medical practitioner. The research cluster of the ALP framework or APF, was an area where they described the need for supervision to enable them to develop effectively to advanced level.

“You have to work with people who already have the knowledge and experience in research so that they can guide you through.”

*(Participant 3, district general hospital, less experienced)*

Some of the pharmacists interviewed highlighted the lack of support structure within the pharmacy profession. This was due to a lack of pharmacists who have undertaken the RPS Faculty assessment. This is partly because the RPS Faculty is relatively new and it is hoped with time this should improve. In the short term, this means there is a lack of suitably trained pharmacists available to support others developing ALP.

“Senior people who are advanced practitioners being available to support. We have got a lot of senior pharmacists out there who haven't been credentialed, who haven’t gone through the process and had an assessment of their skills.”

*(Participant 4, teaching hospital, less experienced)*

### 4.2. Work-Based Learning

In this study work-based learning within the critical care and pharmacy environments was significant in critical care pharmacists’ development of their practice. 

#### 4.2.1. Critical Care

Within the critical care environment, developing ALP was the result of integration into the multi-disciplinary team (MDT), teamwork and exposure to different patient groups, clinical situations and problems.

##### 4.2.1.1. Multidisciplinary Working

A key way in which pharmacists developed their practice was through integration into the critical care MDT. They were able to develop alongside the medical staff by participating in general discussion, ward rounds, teaching sessions and journal clubs. The pharmacists highlighted the importance of joining the medical community of practice in order to receive mentorship, supervision and to develop the knowledge and skills required for ALP. However, the participants highlighted that learning within the medical community of practice does not always provide the appropriate knowledge for critical care pharmacists.

“Unless someone has said to you, ‘You need to know about this and this at this level’. You’re often self-taught and it’s luck of the draw whether you learn useful stuff or whether you learn things that are perhaps more relevant to doctors than pharmacists.”

*(Participant 8, teaching hospital, less experienced)*

##### 4.2.1.2. Teamwork 

Working as part of a critical care pharmacy team was identified to be an advantage to developing ALP offering access to peer and expert critical care pharmacists. This provided these pharmacists with the ability to understand what ALP involved, have a clearly defined development pathway and role models to emulate. The pharmacists were able to learn from the experts within their team; through observation of ALP, discussion of problems and expert assessment and feedback. This ensured that pharmacy specific critical care knowledge and skills were developed within the workplace. The ease of access to advice, support and supervision from both the experts and peers within the critical care team was seen as invaluable.

“I had a lot of support from the critical care pharmacy team, the senior pharmacists, were always available for patient reviews, reviewing charts or going through problems. So from a clinical practice point of view they were there on day-to-day basis to support what I was doing.”

*(Participant 4, teaching hospital, less experienced)*

Overall, both lone practitioners and those working in a critical care pharmacy team identified the benefits of the traditional medical model of training which is underpinned by teamwork and involves experiential learning and apprenticeship [16].

“even three months of a supernumerary type option for pharmacists working at foundation level to rotate to a teaching hospital and work within a critical care pharmacy team. This would give them a huge opportunity and that’s something that is taken for granted by medical staff for example.”

*(Participant 1, teaching hospital, experienced)*

#### 4.2.2. Pharmacy

The pharmacy environment was defined as the uni-professional environment found within the confines of the hospital pharmacy department. This environment offered the critical care pharmacists opportunities to develop professional skills such as leadership, management and education. This was facilitated by interaction with their pharmacy peers who often worked within different clinical specialties.

Peer observation and sometimes discussion with their peers allowed them to reflect on their practice and identify areas for development.

*“When you have got pharmacist colleagues that work in different fields, not critical care, you see them displaying certain qualities of skills that belong to advanced practice and you think they are really good at this, I really should develop more in that direction.”*

*(Participant 3, district general, less experienced)*

However pharmacists’ access to these opportunities was influenced by the ethos and culture of their pharmacy department. Departments with a positive advanced practice ethos; established by pharmacy management promoting ALP, encouraging peer review and aligning job descriptions and appraisals to the APF, enabled critical care pharmacists to develop ALP. Unfortunately, some of the pharmacists interviewed described working in a restrictive pharmacy environment with a lack of support and encouragement to undertake the RPS faculty process.

“At the moment it’s just people doing the RPS Faculty off their own back and not necessarily being supported by the senior pharmacy management, who should be supporting them.”

*(Participant 8, teaching hospital, less experienced)*

### 4.3. Reflective Practice

Reflective practice involves an individual’s capacity to reflect on their own practice to identify professional development needs and gaps in their knowledge [17]. In this study this was supported by pharmacists observing ALP and through receiving expert assessment of their practice.

The critical care pharmacists did use the ALP framework or APF to identify areas of strength and weakness in their own professional development. However, they highlighted the importance of benchmarking their practice against expert critical care pharmacists and their pharmacy peers within the workplace. In particular, visits to other critical care centres with expert critical care pharmacists afforded them the ability to understand what ALP involved. They could benchmark their current level of practice and identify areas to develop.

“With regard to my level of practice, I suppose I got an idea of my gaps by visiting other critical care centres and seeing practitioners that were more developed that I was.”

*(Participant 1, teaching hospital, experienced)*

Observation of peer and expert practice also occurred virtually through online networks such as the UKCPA message board. The pharmacists suggested that the message board allowed them to identify gaps in their knowledge base, which contributed to self-assessment and aligns with research from the medical profession [18].

“There is a lot of interaction between critical care pharmacists working in the UK. We might meet each other at meetings or go through forums. You hear what other people are doing and see their ideas and share from that. It’s even down to the fact someone will write something on a forum that is clearly well reflected and well thought out, you think I need to know more about that.“

*(Participant 5, teaching hospital, experienced)*

#### Expert Assessment

Expert assessment was felt to be invaluable for the critical care pharmacists to undertake reflective practice. Some of the pharmacists interviewed found the feedback they received from the credentialing process, which involved expert review, beneficial. They suggested it reassured them that they were practising at an appropriate level and allowed them to identify areas of strength and weakness. Their views are supported by the other candidates who undertook the credentialing process [19].

Lack of access to assessment and review by expert critical care pharmacists was considered by the interviewees to be a barrier to developing ALP. The need to develop better access to expert review and assessment for all critical care pharmacists was seen as important in order to develop the advanced level practitioners of the future. In addition, many of the pharmacists interviewed discussed the benefits of the medical model in regards to supervision, mentorship and the use of expert assessment and review.

“from a pharmacist’s point of view, compared to doctors, you’re qualified, pointed in a direction of a ward and said off you go, there is no comparison in the level of support for our development and because of that as a profession we struggle.”

*(Participant 1, teaching hospital, experienced)*

## 5. Discussion

Work-based learning was significant in critical care pharmacists’ development of their ALP. This supports the results of a survey of critical care pharmacists by Warin et al. [10] and is typical of other health-care professionals [20,21,22]. Work-based learning was facilitated by two different communities of practices, critical care and pharmacy. It allowed the pharmacists to develop different aspects of ALP. Fuller and Unwin [23] suggest that being part of multiple communities of practice broadens experiences and positively enhances an individuals’ development.

A lack of work-based supervision was identified as a barrier to developing ALP. The pharmacists highlighted its importance in order to learn certain ALP skills such as research and the critical care pharmacy knowledge. In order to support critical care pharmacists’ development the lack of work-based support structure within the pharmacy profession needs to be addressed. To increase the number of pharmacists able to provide supervision the RPS needs to drive the ALP agenda forward by engaging with chief pharmacists and the NHS. In order to motivate practitioners to develop ALP chief pharmacists need to implement drivers including in house mentorship and peer review programmes and by aligning job descriptions and appraisals to the RPS APF. Currently pharmacy departments are encouraged by the NHS to align job descriptions and appraisals to the Knowledge Skills Framework (KSF) [24]. However, a new NHS document underlining the transformation of hospital clinical pharmacy services has recognised the need to develop advanced level practitioners in the workplace [25]. The RPS needs to work with the NHS to encourage all pharmacy departments to use the APF by either aligning it to the current KSF, which has already been completed by Eggleton et al. [26], or by developing standard APF job descriptions and appraisal templates.

To overcome the lack of work-based supervision the pharmacists strategy in this study was to integrate into the MDT. Their integration into the medical community of practice allowed them to develop new skills and or knowledge, through participation with and supervision from the intensivists [27]. However, interacting with doctors rather than other critical care pharmacists was suggested to develop knowledge and skills more relevant to medical professionals. Unfortunately, for critical care pharmacists working in isolation the learning and knowledge they require is often not yet available within their organisation. Therefore, the traditional modes of work-based learning such as observation and participation in work activities suggested by Billett [27] are not sufficient. Lone practitioners had to use expansive learning approaches which allowed them to construct and adapt new concepts into their own practice [28]. Pharmacists working as part of a critical care pharmacy team were seen to be at an advantage having access to expert pharmacists who had already appropriated the critical care pharmacy knowledge and were able to support them in contextualising this knowledge in a more consistent and timely manner [27]. 

Expert assessment of practice was found to be an important strategy in ALP development and was required to support reflective practice. This aligns with the work of Boud [29] who considers that self-assessment should be completed with the guidance of others. Indeed, the medical profession are favouring peer review as a method to identify learning needs [30]. For example core medical trainees are assessed through structured work based assessments which allow a review of their practice and identification of gaps in their development [31,32]. 

The main strategies of work-based learning and reflective practice that critical care pharmacists use to develop ALP were enhanced by interaction with expert critical care pharmacists. In particular, face-to-face access with expert critical care pharmacists, such as observation and assessment of ALP through expert supervision, was felt would enable more timely development of ALP. A significant proportion of pharmacists work as lone practitioners within the critical care speciality and have limited access to expert critical care pharmacists. Increasing face-to-face access with these experts is the most significant area for the UKCPA to develop within their education and training programme. One suggestion to increase face-to-face access with expert critical care pharmacists is to implement a supernumerary work-based learning programme through the development of specialist training centres or regional networks. However, the practicalities of developing this programme with limited funding from an already stretched NHS is likely to be difficult. In addition, there are only limited numbers of expert critical care pharmacists in the UK compared with approximately 400 pharmacists practicing within the critical care speciality [9]. Therefore, identifying ways to implement or improve access to expert critical care pharmacists presents an interesting further research project and will need to involve the UKCPA and RPS collaborating with the NHS.

## 6. Limitations

A key limitation of this study was that all the pharmacists interviewed had achieved ALP status and therefore their views may not be representative of the rest of the UK critical care pharmacists. Further work could involve identifying the views of pharmacists aspiring to become advanced level critical care practitioners. Additionally, the MDT provided a significant amount of support to the critical care pharmacists and gaining their perceptions may also be helpful.

## 7. Conclusions

The UKCPA CCG’s objective is to increase the number of critical care pharmacists practising at advanced level in order to meet the recommendations of UK national standards for Intensive Care Units. The results of this study highlight that to increase the number of MFRPSII level practitioners within critical care support for their ALP development is required. This support involves developing face-to-face access to expert critical care pharmacists within a national training programme. Additionally, chief pharmacists need to implement drivers including:- in house mentorship and peer review programmes and align job descriptions and appraisals to the Royal Pharmaceutical Society, Great Britain, Advanced Practice Framework. It is felt that these strategies would also be applicable and beneficial for the development of ALP for pharmacists working in other clinical specialities. 

## Appendix A. Interview Schedule

### **Brief background**

Firstly I want to ask you some brief background questions;

1. When did you apply for the credentialing process and why did decide to undertake the credentialing process then?
2. Can you give me a brief synopsis of how you became a Critical Care pharmacist working in your current role?
  → Prompt: What were your previous jobs roles, how did this prepare you for your current role?

### **Development of advanced practice**

I now want to ask you some questions about how you developed your practice to advanced level;

1. Can you explain to me how you feel you have developed your practice to advanced level (FSII)?
  → Prompt: For example what things have enabled you to do this?
  → Prompt: For example were there any barriers or challenges to this? If so what were they? And how did you overcome these challenges or barriers?
2. During the process of developing your practice how did you identify any gaps in your knowledge or competency?
  → Prompt: How did you identify gaps in your professional development?
  → Prompt: Did you do anything help you to identify these gaps? Can you give me examples of this? Was this helpful? Any improvements?
  → Prompt: How did you meet gaps in your knowledge/professional development?
3. How did you know you were practicing at advanced level and ready to undertake the Advanced Level accreditation process?
  → Prompt: Can you give me any examples that made you decide you were ready to be assessed?
  → Prompt: Did anybody influence your decision to be assessed?
4. How do you think your work environment has influence the development of your practice?
  → Prompt: Can you give me some examples of how your work environment helped or challenged in developing your practice?
  → Prompt: Are there any areas that the workplace did not develop your practice—can you give examples of this?
  → Prompt: How did you ensure you got the most out of your workplace to develop your practice?
5. Did you have any support to develop your practice? If yes who supported you and in what ways did they support you?
  → Prompt: People within your workplace or outside of the workplace or outside of your profession?
  → Prompt: Did you find this support to be helpful? In what ways?
6. Based on your own experiences, what advice would give me as a foundation level CC pharmacist hoping to develop my practice into Advanced level?
  → Prompt: Are there any aspects of your own approach that I could use to develop my practice?
  → Prompt: What challenges do you feel that critical care pharmacists such as myself face in developing their practice to advanced level?
  → Prompt: How do you think these challenges might be overcome?
  → Prompt: Are there any resources that could be useful to me? Are there any resources that you feel could be beneficial that are not currently available?
